# Transcription Factor Regulation of Gene Expression Network by *ZNF385D* and *HAND2* in Carotid Atherosclerosis

**DOI:** 10.3390/genes15020213

**Published:** 2024-02-07

**Authors:** Ming Tan, Lars Juel Andersen, Niels Eske Bruun, Matias Greve Lindholm, Qihua Tan, Martin Snoer

**Affiliations:** 1 Department of Cardiology, Zealand University Hospital, 4000 Roskilde, Denmark; mingtan91dk@gmail.com (M.T.); laad@regionsjaelland.dk (L.J.A.); nbru@regionsjaelland.dk (N.E.B.); mgl@regionsjaelland.dk (M.G.L.); marsn@regionsjaelland.dk (M.S.); 2 Department of Clinical Medicine, Faculty of Health and Medical Sciences, University of Copenhagen, 2200 Copenhagen, Denmark; 3 Department of Clinical Medicine, University of Aalborg, 9260 Aalborg, Denmark; 4 Epidemiology, Biostatistics and Biodemography, Department of Public Health, Faculty of Health Science, University of Southern Denmark, Campusvej 55, 5230 Odense, Denmark; 5 Unit of Human Genetics, Department of Clinical Research, Faculty of Health Science, University of Southern Denmark, Campusvej 55, 5230 Odense, Denmark

**Keywords:** transcription factors, *ZNF385D*, *HAND2*, regulatory network, gene expression, carotid atheroma

## Abstract

Carotid intima-media thickness (CIMT) is a surrogate indicator for atherosclerosis and has been shown to predict cardiovascular risk in multiple large studies. Identification of molecular markers for carotid atheroma plaque formation can be critical for early intervention and prevention of atherosclerosis. This study performed transcription factor (TF) network analysis of global gene expression data focusing on two TF genes, *ZNF385D* and *HAND2*, whose polymorphisms have been recently reported to show association with CIMT. Genome-wide gene expression data were measured from pieces of carotid endarterectomy collected from 34 hypertensive patients (atheroma plaque of stages IV and above according to the Stary classification) each paired with one sample of distant macroscopically intact tissue (stages I and II). Transcriptional regulation networks or the regulons were reconstructed for *ZNF385D* (5644 target genes) and *HAND2* (781 target genes) using network inference. Their association with the progression of carotid atheroma was examined using gene-set enrichment analysis with extremely high statistical significance for regulons of both *ZNF385D* and *HAND2* (*p* < 6.95 × 10^−7^) suggesting the involvement of expression quantitative loci (eQTL). Functional annotation of the regulon genes found heavy involvement in the immune system’s response to inflammation and infection in the development of atherosclerosis. Detailed examination of the regulation and correlation patterns suggests that activities of the two TF genes could have high clinical and interventional impacts on impairing carotid atheroma plaque formation and preventing carotid atherosclerosis.

## 1. Introduction

As an ultrasound marker of carotid atherosclerosis, carotid intima-media thickness (CIMT) is a measure of the combined thickness of intima and media layers of the carotid arteries. The CIMT has been reported as representative of subclinical and asymptomatic atherosclerotic vascular diseases, and therefore determination of CIMT is a procedure to detect primordial atherosclerosis implicated in the development of cardiovascular diseases (CVD), cognitive impairment, and white matter hyperintensities [1,2,3]. Epidemiologic studies have shown that CIMT can be influenced by multiple factors including genetic and environmental elements like lifestyle factors [4], dietary patterns [5], etc. The genetic contribution to CIMT has been estimated to be 40% in a family study [6]. A twin study reported a heritability estimate of around 60% [7]. The study also revealed that most of this genetic effect occurs through pathways independent of traditional coronary risk factors. The genetic contribution to CIMT has inspired a large number of genome-wide association studies (GWASs) to look for single-nucleotide polymorphisms (SNPs) that influence CIMT [8]. Although multiple SNPs have been detected to associate with CIMT measurements, a very large proportion of its variation remains unexplained. A recent large-scale GWAS based on the UK Biobank data with CIMT measurements identified seven novel loci that were associated with all three phenotypes of CIMT (minimum, mean, and maximum thickness) [9]. Of special note, among the reported novel loci, two are transcription factor (TF) genes *ZNF385D* (rs1553085, rs7628630) and *HAND2* (rs4235201, rs188848834).

Transcription factors recognize specific DNA sequences to control chromatin and transcription regulation, forming a complex system that guides the expression of the genome [10], through either activating or repressing the expression of target genes. Mutated or dysregulated transcription factors represent a unique class of drug targets that mediate aberrant gene expression in disease development [11]. Characterizing the regulatory activities of TFs using transcriptomic data offers the opportunity to uncover their targeting genes and networks, thus helping with the understanding of molecular etiologies of specific diseases. This accumulated knowledge can contribute to the design of interventional and treatment strategies.

The TF gene *HAND2* is a vascular development-associated gene. Its regulatory targets have been found to control mesenchymal transition underlying cardiac cushion development in the atrioventricular canal [12]. A recent study found that a baseline expression of the gene in the adult heart is required to withstand right ventricular pressure overload [13]. Variation of the *ZNF385D* gene (rs13070110) was nominally associated with an increased risk of intracerebral hemorrhage [14]. *ZNF385D* may influence several of the negative symptoms of schizophrenia according to a meta-analysis of two genome-wide association studies [15]. Moreover, elevated expression of *ZNF385D* may be associated with anxiety and depressive symptoms that often occur in patients with chronic obstructive pulmonary disease [16]. As transcription factors, the regulatory patterns of the two genes in the above-mentioned diseases as well as in CIMT, have not been investigated.

This study applies transcriptional regulatory network analysis [17] to genome-wide gene expression data on CIMT to infer the target genes (networks) regulated by *ZNF385D* and *HAND2*. Furthermore, the joint association with CIMT is explored to obtain a better understanding of TF involvement in carotid atheroma plaque formation and explore its impacts on the intervention and prevention of carotid atherosclerosis.

## 2. Materials and Methods

### 2.1. Study Samples and Transcriptomic Data

This study uses global gene expression data contributed in 2013 and last updated in 2018 by Catherine Cerutti and colleagues at Université Lyon 1 and Hôpital Nord-Ouest to the Gene Expression Omnibus with accession number GSE43292. Detailed information about sample collection and laboratory experiments can be found elsewhere [18]. In brief, 34 patients (5 females and 29 males, mean age 70 years) who underwent carotid endarterectomy were included in the study. The carotid endarterectomy samples were collected in the surgery room and immediately dissected into two fragments: Atheroma plaque (ATH) of stages IV and above according to the Stary classification, containing the core and shoulders of the plaque, each paired with one sample of distant macroscopically intact tissue (MIT) of stages I and II. Genome-wide gene expression data were obtained using the Affymetrix GeneChip Human Gene 1.0 ST arrays (Affymetrix, Santa Clara, CA, USA) covering a total of 764,885 distinct probes from 20,267 genes. Before data analysis, the probe-level expression data were first log-transformed with base two and then averaged across multiple probes within each gene to obtain gene-level expression data.

### 2.2. Transcriptome-Wide Association Study (TWAS)

To perform the network-based analysis for the cluster of targeted genes of a specific TF, each gene covered by the platform needs to be statistically tested to provide a basic distribution for the subsequent enrichment testing of a specific TF-regulated network. Considering the self-matched experiment design, a paired t-test was applied to compare the mean expression level in atheroma plaque (ATH) with that from the macroscopically intact tissue (MIT) for each gene. A genome-wide significance was defined by an adjusted *p* < 2.5 × 10^−6^ using the Bonferroni correction.

### 2.3. TF Regulatory Network Inference

There have been multiple software packages for TF regulatory network analysis published in the literature. Here we applied a well-maintained and popular Bioconductor package, RTN 2.26.0 (Reconstruction of Transcriptional regulatory Networks and analysis of regulons, https://bioconductor.org/packages/devel/bioc/vignettes/RTN/inst/doc/RTN.html) (accessed on 15 January 2024) for TF regulatory network analysis. By defining the set of genes controlled by a given TF as a regulon, the RTN package provides classes and methods for the reconstruction of TRNs and analysis of regulons. The network inference procedure starts with computing MI (mutual information) [19] between a regulator and all potential targets using function tni.constructor() and removing non-significant associations by permutation using function tni.permutation(), followed by additional steps that remove unstable and weak interactions in any triplet formed by two TFs and a common target gene. 

### 2.4. TF Regulatory Network Analysis

In order to test whether an inferred regulon is positively or negatively associated with CIMT, we applied the gene-set enrichment analysis (GSEA, see more details in Functional Annotation section below) using tna.gsea1() function for one-tailed GSEA (GSEA-1T) and tna.gsea2() for two-tailed GSEA (GSEA-2T). GSEA-1T finds regulons associated with CIMT represented by a ranked list of genes generated from a global differential gene expression signature (i.e., TWAS). Here the regulon’s target genes are considered a gene set, which is evaluated against a phenotype (here the absolute value of log fold change, logFC, with base 2). The GSEA-1T uses a rank-based scoring metric to test the association between the gene set and CIMT [20]. A GSEA-2T considers the target genes could be either up (or positively) or down (or negatively) regulated by a TF. The function first maps the target genes in a regulon to the distribution of all ranked TWAS genes by phenotype (logFC). The algorithm in both GSEA-1T and GSEA-2T calculates an enrichment score (ES) that reflects the degree to which the target genes are overrepresented at the extremes (top or bottom) of the entire ranked distribution. Note that GSEA-2T calculates two ESs, ESpositive, and ESnegative, for positively or negatively regulated target genes, respectively, with the difference between them (ΔES = ESpositive − ESnegative) representing the overall regulon activity. Finally, the statistical significance of a regulon is assessed by a permutation test with 1000 random permutations or replications. A permutation *p* < 0.05 is considered statistically significant.

### 2.5. Functional Annotation

To test if specific biological pathways or gene sets are enriched by the member genes of a constructed regulon, we performed GSEA using the R package clusterProfiler 4.10.0 [21]. The package offers two distinct approaches for testing pathway significance, i.e., the over-representation analysis (ORA) and gene set enrichment analysis (GSEA) [20]. ORA is based on comparing the overlap between two gene sets with the expected overlap by chance, using the hypergeometric test which is basically a contingency table analysis. GSEA, on the other hand, ranks all genes in the dataset based on their differential expression and then tests whether genes in a gene set are enriched at the top or bottom of the ranked list as reflected by an enrichment score. While ORA focuses on the overlap between gene sets, GSEA takes into account the entire distribution of gene expression changes. We opted to apply GSEA instead of ORA for our functional annotation of the two regulons because the former makes use of more information from the data in the statistical testing. Following the procedures in the package, we estimated an enrichment score for each pathway and then assessed its statistical significance using a permutation test with 1000 random replicates. The enrichment analysis step took account of the ranked statistics of member genes (t statistic from TWAS) in estimating the enrichment score. Our functional annotation analysis focused on biological pathways from the Kyoto Encyclopedia of Genes and Genomes (KEGG). The significance of KEGG pathways was defined upon correction of multiple testing using the adjusted *p* value or FDR < 0.05.

## 3. Results

### 3.1. Global Differential Expression Analysis

**Figure 1:**
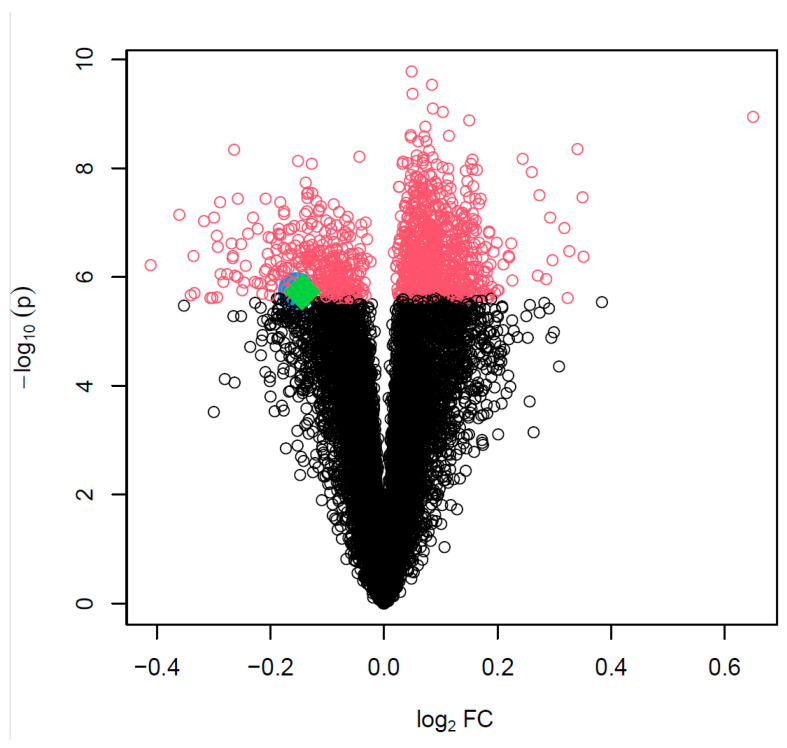
Volcano plot plotting statistical significance (minus log *p* value with base 10) against effect size of each gene tested in the global differential gene expression analysis. The 1111 genes reaching genome-wide significance with *p* < 2.5 × 10^−6^ are marked with red color. The two transcription factor genes are plotted as large symbols with *ZNF385D* colored green (filled diamond) and *HAND2* colored blue (filled circle).

To provide a reference distribution of statistical testing on differential expression of the 20267 genes for subsequent network enrichment analysis, we first performed a global gene expression analysis i.e., TWAS using a paired t-test. After adjusting for multiple tests using the stringent Bonferroni correction, 1111 genes meet genome-wide significance with *p* < 2.5 × 10^−6^. Figure 1 is a volcano plot displaying the statistical significance (minus log *p* value with base 10) against the fold change (log scale with base 2, logFC), with the 1111 genes colored red (Supplementary Table S1). The two transcription factor genes, *ZNF385D* (logFC = −0.14, *p* = 1.88 × 10^−6^) and *HAND2* (logFC = −0.15, *p* = 1.82 × 10^−6^), are specifically shown by enlarged symbols (*ZNF385D* as red, *HAND2* as blue) in Figure 1. Both genes are down-regulated in ATH with logFC < 0 and with nearly borderline genome-wide significance.

### 3.2. Inference of Transcriptional Network

Transcriptional network inference for *ZNF385D* identified a large and balanced network with a total of 5644 target genes, among them 3078 genes are positively regulated, and 2566 genes are negatively regulated by *ZNF385D* (Table 1). For *HAND2*, a large but unbalanced regulatory network was detected, consisting of 781 target genes—144 with positive and 637 with negative regulation by *HAND2*. This network is unbalanced because there are many more target genes that are negatively regulated.

### 3.3. Analysis of Transcriptional Network

**Figure 2:**
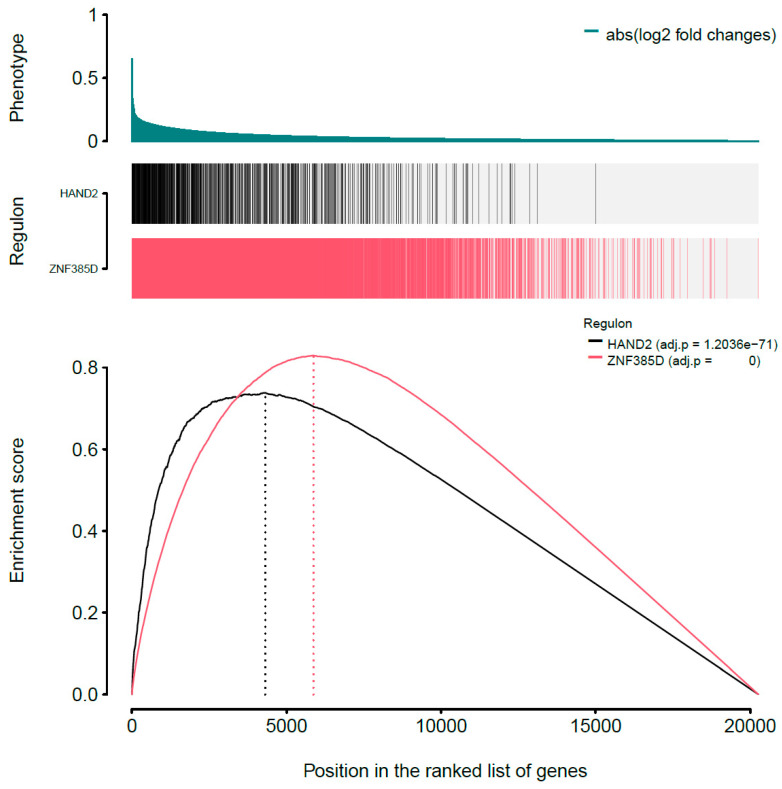
One-tailed GSEA analysis showing genes in each regulon (vertical bars) ranked by the phenotype as absolute logFC from global differential gene expression analysis. Both *ZNF385D* and *HAND2* regulons are extremely significantly enriched for high effect sizes in their association with CIMT.

**Figure 3:**
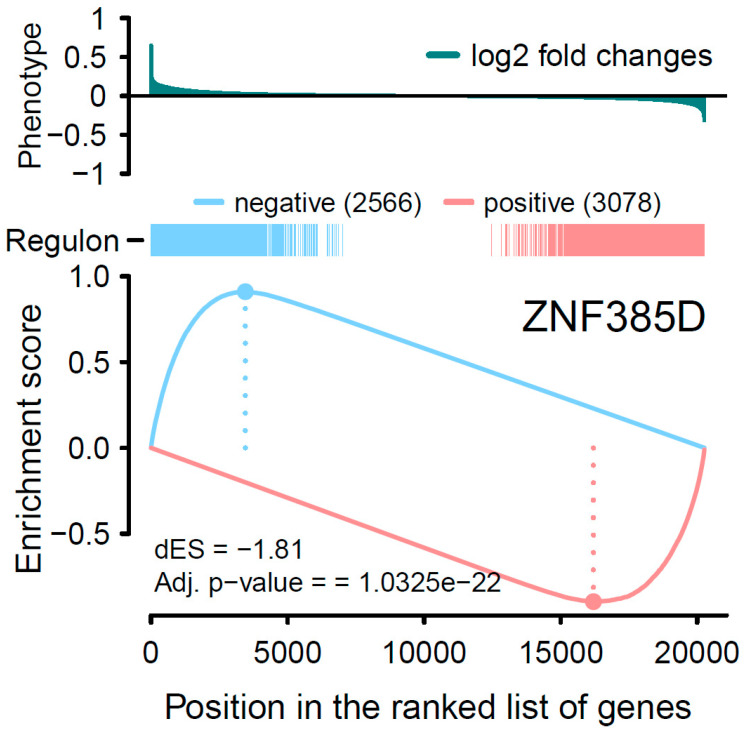
Two-tailed GSEA analysis showing *ZNF385D* regulon’s positive/negative targets (red/blue vertical bars) ranked by the phenotype as logFC from global differential gene expression analysis. The positive (red) and negative (blue) regulation target genes are significantly enriched for decreased and increased expression patterns in ATH, respectively.

**Figure 4:**
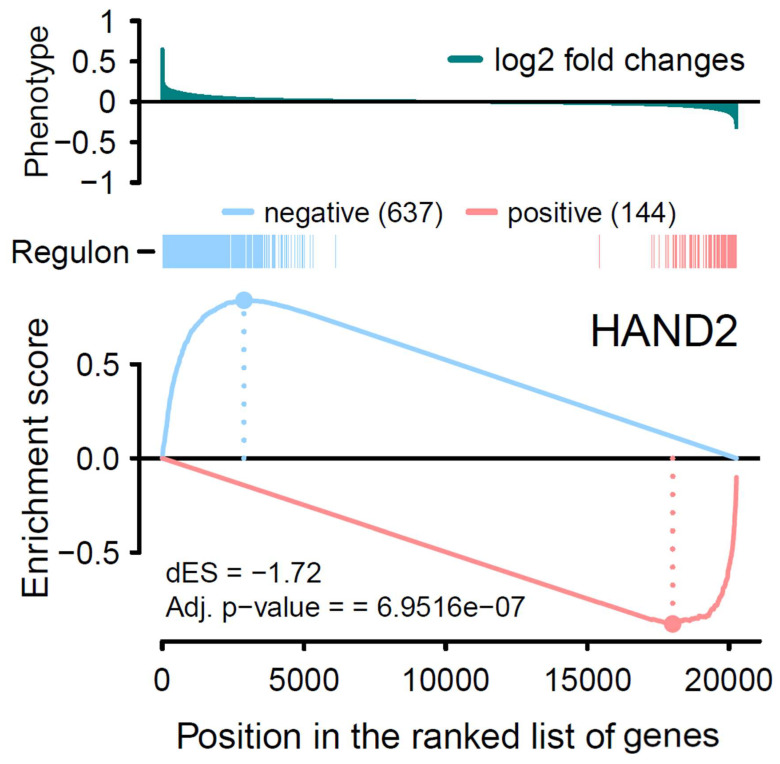
Two-tailed GSEA analysis showing *HAND2* regulon’s positive/negative targets (red/blue vertical bars) ranked by the phenotype as logFC from global differential gene expression analysis. The positive (red) and negative (blue) regulation target genes are significantly enriched for decreased and increased expression patterns in ATH, respectively.

By introducing GSEA, we first started with the one-tailed test, GSEA-1T, where genes are ranked by their phenotype or effect size (absolute logFC). For *ZNF385D*, an enrichment score of 0.83 is observed with *p* < 2.26 × 10^−308^ (Table 1, Figure 2). For *HAND2*, the ES is estimated as 0.74 with a *p* value of 1.2 × 10^−71^. In Figure 2, the target genes in both networks (regulons) are densely mapped to the left side of the ranked phenotype (absolute logFC) distribution. As a result, both networks are extremely significantly enriched by genes differentially expressed in ATH. We next move to a two-sided test, GSEA-2T, taking the direction (positive and negative) of TF regulation into account. For *ZNF385D*, the ES for positive regulation, ES_positive_, is -0.9 (*p* = 1.18 × 10^−20^), and that for negative regulation, ES_negative_, is 0.91 (*p* = 3.03 × 10^−7^) (Table 1). The difference between positive and negative enrichment scores, ∆ES, which represent the overall activity of the network, is 1.81 with a *p* value of 5.16 × 10^−23^. It can be seen from Figure 3, that the target genes in the network are enriched at two ends of the phenotype distribution of ranked logFC showing both increased (negatively regulated by *ZNF385D*, colored blue) and decreased (positively regulated by *ZNF385D*, colored red) expression in ATH. The figure also displays an adjusted *p* value of 1.03 × 10^−22^ for the overall regulatory activity of the network because two networks have been tested. Likewise, GSEA-2T on the *HAND2* regulated network estimated an ES_postive_ of -0.88 (*p* = 2.64 × 10^−4^), an ES_negative_ of 0.84 (*p* = 4.4 × 10^−4^), and an ∆ES of −1.72 (*p* = 6.95 × 10^−7^) (Table 1). The unbalanced regulation can be seen in Figure 4, where many more target genes are mapped to the left side of the ranked phenotype distribution.

### 3.4. Enriched KEGG Pathways by TF Target Genes

**Figure 5:**
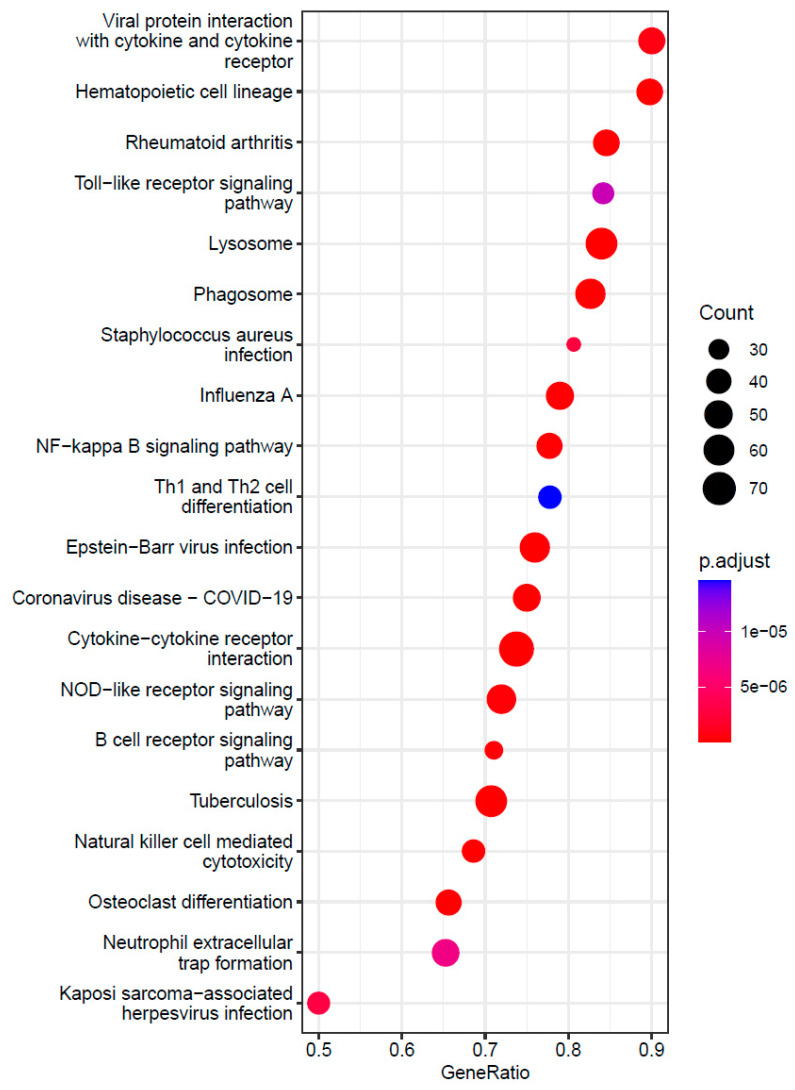
A dot-plot displaying the top 20 most significantly enriched KEGG pathways by the member genes of *ZNF385D* regulon. Each pathway is plotted against corresponding geneRatio which is the ratio of input genes that are annotated in a pathway.

In order to interpret the biological function of the genes in each regulon, we extracted the IDs of target genes in a regulon and their test statistics from TWAS (Supplementary Tables S2 and S3). We then performed gene-set enrichment analysis of biological pathways in the KEGG database using clusterProfiler. For the large *ZNF385D* regulon of 5644 target genes, our analysis found 107 significantly enriched pathways with adjusted *p* value or FDR < 0.05 (Supplementary Table S4), among them 67 pathways with FDR < 0.01. Interestingly, among the 67 very significant pathways, all are positively enriched (normalized enrichment score NES > 0) suggesting up-regulation or activation of the member genes in each pathway. Figure 5 displays the top 20 significant pathways arranged by their geneRatio (ratio of input genes that are annotated in a pathway). Among the top significant pathways are lysosome, phagosome, and osteoclast differentiation but nearly all pathways are related to the immune system’s response to inflammation and infection including bacteria (tuberculosis), virus (Epstein-Barr virus, COVID-19, influenza-A, measles, hepatitis viruses, etc.), and other antigens (malaria). The significantly enriched pathways also cover multiple autoimmune diseases, e.g., rheumatoid arthritis, autoimmune thyroid disease, type I diabetes mellitus, and asthma. For the small regulon of *HAND2* with 781 target genes, only one KEGG pathway was found significantly enriched, the chemokine signaling pathway. The pathway is likewise positively enriched with FDR = 3.65 × 10^−2^. As chemokine signaling is essential for coordinated cell migration in health and disease to specifically govern cell positioning in space and time, the result again emphasizes the role of immune activity in association with atheroscleropathy.

## 4. Discussion

Based on high coverage global gene expression data, we can infer and test the transcriptional networks of two transcription factors, *ZNF385D* and *HAND2*, whose genetic polymorphisms have been very recently reported to show association with carotid intima-media thickness in a large-scale GWAS [9]. As a transcriptomic approach, our results provide novel insights concerning the regulatory mechanism of the two TFs in the development of carotid atherosclerosis while suggesting the critical impact of TF regulation in cardiovascular pathogenesis.

It is important to note that, in Figure 1, the expression activities of both *ZNF385D* and *HAND2* are downregulated in the diseased tissue (ATH). From Figure 3 and Figure 4, it is clear the target genes that are activated in ATH show a negative correlation with *ZNF385D* and *HAND2* activities, while target genes that are suppressed in ATH are positively correlated with the functionalities of the two TFs. In other words, the target genes in the TF networks that are active in ATH are inhibited by TF expression, while target genes that are suppressed in ATH are activated by TF expression. Such observation can have high clinical and interventional relevance, as activation of the two TF genes could lead to altered expression patterns unfavorable to carotid atheroma plaque formation and potentially slow down or stop carotid atherosclerosis.

As genetic polymorphisms of the two TFs genes are reported to associate with CIMT [9], the extremely high significant expression patterns of their inferred regulatory network could together suggest that the significant SNPs from the GWAS function as expression quantitative loci (eQTL) in the pathogenesis of CIMT. Here the effective variants of the significant SNPs could manifest improved binding affinity to target genes to activate or inhibit their transcription [22]. Although our network-based analysis for *ZNF385D* and *HAND2* showed an extremely high significant association with CIMT, as can be seen from Figure 1, the two genes are not on top of the most significant genes in TWAS. We thinkthat the different polymorphisms at the relevant SNPs could mean that only carriers of the effective alleles benefit from the favorable regulation patterns of the network, while what we see from Figure 1 is an overall mean expression of both carriers and non-carriers in the samples. Further studies collecting SNP and gene expression data on the same individuals should help to clarify the relationship in eQTL regulation.

It is generally believed that lifestyle risk factors for atherosclerosis are also partly genetically determined and some of the variants, which play a role in atherogenesis overlap with those modulating its risk factors [23]. However, a traditional genetic epidemiology study using twin modeling indicated that most of this genetic effect on CIMT occurs through pathways independent of traditional coronary risk factors [7]. The extremely strong CIMT association from our inferred gene expression networks under the regulation of genetically polymorphic *ZNF385D* and *HAND2* is in support of genetically controlled functional pathways independent of lifestyle and behavior factors in the development of atherosclerosis.

Biological studies of the two TF genes, *ZNF385D* and *HAND2* on their functional involvement in atherosusceptibility are extremely sparse [9,24]. In this regard, our pathway analysis of the two regulons provides a novel approach for the functional investigation of transcription factors when their regulatory networks can be established. In our enrichment analysis of KEGG biological pathways, the significant pathways enriched by the two regulons are heavily dominated by immune response to inflammation and infection suggesting strong immunity-related functional involvement of the two TF genes. A recent epigenetic study on post-COVID-19 patients found a significantly differentially genomic region located in the vicinity of the *ZNF385D* gene by comparing severe versus mild COVID-19 patient groups (*p*  =  4.82 × 10^−14^). Another study reported that *HAND2* plays a role in cellular inflammation and immune system function [25].

Multiple clinical epidemiological studies have shown a positive correlation between CIMT and the level of C-reactive protein which is a type of protein associated with inflammation in the body [26,27,28]. However, the lack of causality in the correlation [29] suggests the existence of causal pathological conditions that need to be considered. In a recent study, Çırakoğlu and Yılmaz [30] reported a positive association between the systemic immune–inflammation index and CIMT in hypertensive patients. Likewise, Veldhuijzen van Zanten and Kitas [31] found an increased CIMT in patients with rheumatoid arthritis and suggested the importance of the intensity of inflammation in states of high-grade systemic inflammation. Increased CIMT was also observed in patients with HIV [32] and inflammatory bowel disease [33]. Alfaddagh et al. [34] pointed out that although inflammation is in general beneficial and has evolved to promote survival, chronically activated and sustained inflammation can incite progressive tissue injury and eventually result in reduced survival. Mounting scientific and clinical evidence demonstrates that every step of atherogenesis, from the development of endothelial cell dysfunction to foam cell formation, plaque formation and progression, and ultimately plaque rupture stemming from architectural instability, is driven by the cytokines, interleukins, and cellular constituents of the inflammatory response [34]. This notion is replacing the traditional view of atherosclerosis as a disease of passive cholesterol accumulation in the subendothelial space. The significant enrichment of immunity and inflammatory response pathways in our GSEA analysis of the identified TF-regulated networks provides novel evidence based on transcriptomic data that support the critical role of chronic inflammation in the development of CIMT. Our result merits further experimental investigations so that the causal relationship between chronic inflammation and CIMT can be established.

In Figure 2, the one-sided GSEA obtained much higher statistical significance as indicated by the extremely low *p* values (close to 0). This enriched power in statistical testing is because GSEA-1T jointly tests both positively and negatively regulated target genes while ignoring the direction of TF regulation. As exemplified in our analysis, it is; however, highly important to test the enrichment patterns of the positive and negative regulations in the network, so that a more insightful interpretation of the TF activity in disease pathogenesis can be made and clinical or interventional impacts discussed. To this end, it is sensible to perform both one and two-sided GSEAs for evaluating each inferred transcriptional network.

Overall, our analysis of the transcriptional networks of *ZNF385D* and *HAND2* reveals extremely high statistical significance in their joint contribution to CIMT development. Detailed examination of the regulation and correlation patterns suggests that the regulatory activities of the two TF genes could have high clinical and interventional impacts on retarding and preventing carotid atherosclerosis and cardiovascular diseases.

## Figures and Tables

**Table 1:** Test results of network inference and enrichment analysis of ZNF38D5 and HAND2.

	*ZNF38D5*	*HAND2*
Network inference		
Regulon size	5644	781
Positive regulation	3078	144
Negative regulation	2566	637
Enrichment analysis		
GSEA one-sided		
ES *	0.83	0.74
*p* value	<2.26 × 10^−308^	1.20 × 10^−71^
GSEA two-sided		
Positive enrichment		
ES_positive_	−0.90	−0.88
*p* value	1.18 × 10^−20^	2.64 × 10^−4^
Negative enrichment		
ES_negative_	0.91	0.84
*p* value	3.03 × 10^−7^	4.4 × 10^−4^
Differential		
∆ES *	−1.81	−1.72
*p* value	5.16 × 10^−23^	6.95 × 10^−7^

* ES: Enrichment Score; ∆ES = ES_positive_ − ES_negative_.

## Data Availability

Publicly available datasets were analyzed in this study. This data can be found here: https://www.ncbi.nlm.nih.gov/geo/ (accessed on 15 January 2024) with accession number GSE43292.

## Supplementary Materials

The following supporting information can be downloaded at: https://www.mdpi.com/article/10.3390/genes15020213/s1, Table S1: Results of TWAS; Table S2: *ZNF385D* target genes and test statistics; Table S3: *HAND2* target genes and test statistics; Table S4: GSEA results on_KEGG_pathways for regulon *ZNF385D*.
